# The Relationship between Early Word Reading, Phonological Awareness, Early Music Reading and Musical Aptitude

**DOI:** 10.3390/jintelligence10030050

**Published:** 2022-08-03

**Authors:** Márta Janurik, Noémi Surján, Krisztián Józsa

**Affiliations:** 1 Béla Bartók Faculty of Arts, University of Szeged, 6722 Szeged, Hungary; gevayne.janurik.marta@zmk.szte.hu; 2 Doctoral School of Education, University of Szeged, 6722 Szeged, Hungary; noemi.surjan@gmail.com; 3 Institute of Education, University of Szeged, 6722 Szeged, Hungary; 4 Institute of Education, Hungarian University of Agriculture and Life Sciences, 7400 Kaposvár, Hungary

**Keywords:** early music reading, early reading, auditory processing, phonological awareness

## Abstract

A wide range of evidence has demonstrated the impact of music learning on phonological awareness and the development of reading. Music reading, its relationship with linguistic abilities and reading skills are all highly researched areas. However, limited information is available regarding the relationship between early text reading and early music reading. This study examined the relationship between word reading and music reading, musical aptitude and phonological awareness. The sample consisted of 119 Hungarian grade 1 elementary school students, who were at the beginning of both their text-reading and music-reading studies. They had commenced their studies just nine months before the point of assessment. Phonological awareness, musical aptitude and music reading were the independent variables in the linear-regression model, whilst word reading was the dependent variable. Together, the independent variables explained 50% of the level of development of word reading. The findings suggest a link between early word reading and early music reading. Moreover, further research as well as transfer research may benefit from looking at the possible effects of acquiring and practicing symbol reading, a process most frequently accompanying music learning, on the development of text reading.

## 1. Introduction

Correlational and experimental studies have tended to confirm that learning to read may be facilitated by other, indirectly related learning processes such as music learning (e.g., Butzlaff 2000; Corrigal and Trainor 2011). Learning musical instruments as well as music programs used in studies that include improvisation or playing an instrument often require music reading or the use of simple musical notations. Previous findings have demonstrated that the two types of reading, which are based on auditory processing, that is, learning to read musical notations and learning to read words, involve many of the same processes (e.g., Forgeard et al. 2008b). This suggests that the development of music reading and text reading may be linked, especially at the beginning of their acquisition process. However, a relatively limited number of empirical studies have examined the possible links between early music reading and early text reading. In part, this is probably because within different education systems, learning the two skill sets rarely begins at the same time, as there are differing views on the process of auditory and cognitive development that establish music reading as well as the role of music reading in musical thinking. For example, in Gordon’s (1997) skill-learning-sequence model, learning to read music is included only in the final level (Theoretical Understanding), and the Orff and the Suzuki methods both assume that early music reading inhibits creativity. However, other researchers have emphasized the role of music reading in musical thinking and musical development. Graphic representation of music facilitates the development of musical abilities, and music literacy adds new aspects to the systematization of musical knowledge (Bamberger 1991, 1996; Gromko and Poorman 1998). In Kodály’s music pedagogy, and, thus, in the Hungarian education practice—both in the Hungarian public education system and in the process of learning to play instruments—music reading has a central role from the beginning. The first steps to music reading are already present in the curriculum of preschools in Hungary (Forrai 1998), and children start to learn the system of musical notations from grade 1 (that is, at the age of 6) of elementary school, simultaneously with text reading. This study examined if there are benefits to the simultaneous learning of the two types of literacy, and the possible links of learning to read these two symbol systems.

## 2. Theoretical Framework

### 2.1. Music Reading and Music Processing

Music reading is a process that involves converting specific visual symbols into sounds (Hodges and Nolker 2011). “From a cognitive perspective, music reading requires several simultaneous processes including coding of visual information, motor responses and visual-motor integration” (Gudmundsdottir 2010, p. 2). Schön et al. (2002) distinguish between three ways of transcoding visual symbols: (1) playing-like (visual to motor transcoding); (2) singing-like (visual to auditory transcoding); and (3) naming-like notes (visual to verbal transcoding). Certain cognitive operations and neural networks are the same, while others may well differ. This model assumes a strong link between music writing and reading. Based on the results of studies into brain damage, Hébert and Cuddy (2006) offered another possible alternative, in which the name of the written music note is linked with the motor code.

Music reading is a complex activity that relies on a range of processing abilities. Some of these are associated with perceptual or pattern recognition in the rapid and relatively automatic perception of musical structures, while others are linked to a general knowledge of musical structures (Sloboda 1976, 1984; Waters et al. 1998).

On the level of acoustic analysis, music processing involves the operation of two parallel subsystems, pitch-based processing (the melodic contour and the tonal functions of the successive pitch intervals) and time-based or temporal processing (metric organization and rhythmic structure of the successive durations). These two subsystems are independent of each other (Peretz 2009; Peretz and Coltheart 2003). Music-reading studies have also confirmed that pitch and timing are perceived separately (Gudmundsdottir 2010; Schön and Besson 2002; Waters et al. 1998).

The key prerequisites of music reading are internal music representations that are based on musical perception and are relevant from the aspect of both music reading during playing an instrument and music reading during singing. In the case of singing, the internal representation of pitch and pitch relationships are of particular importance (Fine et al. 2006). Audiation, the internal analog of aural perception as defined by Gordon (1997), may be considered as a cognitive system that facilitates the development of music reading. Gordon uses the term notational audiation in the context of interpreting music reading. It is a specific skill of “hearing”, which refers to the internal hearing of music seen in notation as well as its significance. This is in parallel with Kodály’s concept of inner hearing, which is the auditory image which exists in our minds even without acoustic input (Forrai 1998). Kodály believed that the enhancement of inner hearing is the final aim of all professional music study (Kodály 1974).

### 2.2. Reading, Phonological Awareness and Musical Development

Music and speech perception are the two highest level processes in hearing. Findings of neuropsychology suggest that during language and music processing, certain brain networks specialize in musical functions, while language and environmental sound processing are distinct from each other (Peretz 2009). Other researchers, however, have found overlap in the areas of brain activation during music and language processing (e.g., Tillmann 2012; Wong et al. 2007), and studies with musicians have confirmed different and increased language performance by musicians (Moossavi and Gohari 2019). To reconcile the contradictory evidence, Patel and Iversen (2007) offer a resource-sharing framework, which is particularly relevant for children. This framework assumes common auditory-processing mechanisms for music and language, which may form the basis of the identified relationship between music processing and the phonetic, phonological and prosodic processing of speech.

Music learning involves the development of auditory perception, which may enhance speech recognition in early childhood (Costa-Giomi 1999; Tierney and Kraus 2013). Most of the evidence available about the role of perceptual language abilities in reading acquisition concerns the relationship between phonological awareness and musical abilities. The ability to consciously access the internal structure of words (syllable, sound) enables one to recognize, identify and manipulate phonological units (Gillon 2004; Ziegler and Goswami 2005). It is an important predictor of reading, which is associated with speech sounds and syllables as part of metalinguistic awareness (Deacon 2012; Milankov et al. 2021). On the word level, it means analyzing and manipulating phonological units (rhyme discrimination, syllables), and it is part of the natural process of development. On the phoneme level, it means isolating, segmenting, synthesizing and manipulating sounds. These are conscious activities, and their development is facilitated by learning to read (Ziegler and Goswami 2005). Several studies have confirmed the impact of music learning or that of an advanced level of pitch and/or rhythmic processing on phonological awareness (Anvari et al. 2002; Degé and Schwarzer 2011; Forgeard et al. 2008b; Holliman et al. 2010; Moreno et al. 2011; Overy et al. 2003).

The development of reading is a highly researched area. On the first level, three cognitive abilities, namely phonological awareness, phonics and rapid automatized naming (RAN), play a crucial role in its early development (Ziegler and Goswami 2005). On another level, comprehension enables access to information, its decoding, use and interpretation as well as the ability to reflect on it (Stevens et al. 2019). According to reading research, the essential elements are recognition of letters, phonemic coding, mental lexicon, parsing, generating propositional representations, identifying topics and thematic structures, creating mental models, interpreting the intentions of the author and identifying the genre of the text (Graesser et al. 1997; Stevens et al. 2019).

Several studies have identified the positive impact of music training on reading acquisition (e.g., Butzlaff 2000; Forgeard et al. 2008a). However, these results are relatively controversial compared to the results on phonological awareness (e.g., Lukács and Hombolygó 2019; Swaminathan and Schellenberg 2018). Butzlaff (2000) has described two meta-analyses. According to this description, studies have found that music training and reading abilities are associated positively. However, experimental studies have not found a significant correlation. Therefore, Schellenberg and Weiss (2013) propose that the impacts of music training may be mediated by, amongst other things, perceptual language abilities. The evidence that music training has a causal effect on reading does not seem to be strong enough, so the causal effect between music lessons and phonological awareness is more compelling.

### 2.3. Music Reading and Text Reading

The parallel between music and language constitutes the basis of research on the relationship between music reading and text reading. Commonalities are traceable even on the level of speech and music processing, which are the basis for text reading and music reading, respectively. In addition, researchers typically focus on the relationship between the coding mechanisms of the two notation systems as well as the similarities in their acquisition. Music and language are two hierarchical systems of which the smallest units are sounds: musical sounds and speech sounds, respectively. Through modifying their acoustic characteristics, and encoding their temporal structure, these sounds are then endowed with meaning. The desired communicative function is achieved by the manipulation of the same acoustic characteristics—pitch, time-span, intensity, tone—both in speech and music (Besson et al. 2011). It has been suggested that learning to read musical notation involves many of the same processes as learning to read words. Understanding that written notation proceeds from left to right, recognizing visual patterns, and understanding that visual symbols map on to particular sounds may play a role in the acquisition of reading as well (Forgeard et al. 2008a).

### 2.4. Characteristics of Music Reading/Writing and the Grade 1 Curriculum of Music Pedagogy in Hungary

Students start to learn musical notations at the age of 6 (grade 1) in the Hungarian education system. Musical cognitive processes, the forming of mental representations, are facilitated by enhancing singing skills and musical perception. In practice, this refers to singing, singing games and rhythmic games (Choksy 1981; Dobszay 1972). These activities promote children’s conceptual development as well as their application of already-known concepts (e.g., slow/fast, high/low, soft/loud) to musical sound patterns. Once they have mental representations formed about a specific musical phenomenon, children are taught solmization syllables and rhythmic value symbols (name, symbol). Systematic observation through listening discrimination always precedes learning new material (Turmezeyné Heller et al. 2005).

Learning solmization starts with the semi-ditone (mi–sol), followed by *la*, *do*, and *re,* one by one. Once children are confident in using these syllables, as specified in the curriculum of grade 1 of elementary school, *fa* and *ti* are introduced. In grade 1, students get to know the quarter note and the eighth note as well as the fourth interval and the eighth interval. Rhythm is introduced through listening, clapping, rhythmic repetition of the names of rhythm units, singing and physical exercises. Learning how to write them down comes later (Choksy 1981).

## 3. Objectives

Earlier research suggests that phonological awareness is a robust predictor of reading. The positive impact of music learning on the development of phonological awareness has also been confirmed, and there is evidence to suggest a positive role in reading acquisition as well. Family background, especially parents’ education, also plays a role in the early level of development of both reading and musical abilities. Music learning—especially learning musical instruments or participating in music-intervention programs, as typically used in transfer research—requires the acquisition and active use of music reading to some extent. Previous results suggest that learning to read musical notations involves many of the same processes as learning to read words. However, examining the possible links of learning these two different forms of communication simultaneously has, so far, received limited attention.

Therefore, building on the associations discussed above, we aimed to explore the relationship between early word reading and early music reading at the beginning of the acquisition process. Our research focused on the connections of phonological awareness, which has already been confirmed to be an important linguistic ability in reading acquisition, mothers’ level of education, musical aptitude (musical-perception and reproduction abilities and pitch-related and rhythm-related abilities) and music reading. Using linear-regression models, where the above listed variables were the independent variables, we examined whether the predicting role of music reading and musical aptitude can be established, besides the previously confirmed associations of reading with phonological awareness, family background and musical abilities.

Our research questions were as follows: (1) What is the relationship between musical aptitude (perception, reproduction, pitch-related and rhythm-related abilities), phonological awareness, word reading, music reading and mothers’ education? (2) To what extent does phonological awareness explain the level of development in word reading? (3) To what extent do musical aptitude and its sub-tests increase the explained variance of word reading? (4) Can the predicting role of music reading be established in addition to phonological awareness and musical aptitude?

## 4. Methods

### 4.1. Participants

Participants included 119 Hungarian students (62 boys and 57 girls) from six elementary schools in a city in the southern part of Hungary. The students’ mean age was 6 years and 6 months (*M* = 90.86, *SD* = 3.67 months). All children were monolingual, native speakers of Hungarian with no known hearing or neurological deficits, attentional deficit disorders or reading disabilities. Participants started their text reading and music reading studies at the beginning of the school year. Data collection took place at the end of the school year, in May 2018. Parents gave their written consent to assess their children and record their data.

### 4.2. Instruments

#### 4.2.1. Phonological Awareness

The phonological awareness test (Szili 2016) consisted of 45 items. The test battery was based on the 5000 most common Hungarian words that are required to understand a text one reads in Hungarian. It measures the ability to access the internal sound structure of words on the level of phoneme and phonology. Items were click-based multiple-choice questions, in which students were required to click on images. Each sub-test consisted of 5 items.

Sub-tests covered four areas of phonology: (1) syllable deletion; (2) syllable segmentation; (3) rhyme discrimination in words; and (4) rhyme discrimination at the end of sentences. On the level of phoneme, the sub-tests covered five areas: (5) isolation of speech sounds; (6) synthesis of speech sounds; (7) analysis of speech sounds; (8) discrimination of long-/short-duration speech sounds; and (9) manipulation of speech sounds.

#### 4.2.2. Word Reading

The word reading test (Magyar and Molnár 2015), which consisted of 85 items, was based on the instrument developed originally by Nagy (2004). Items were closed-ended (yes/no); one point was awarded for each correct answer. The sub-tests measured the following areas of word reading: (1) *headwords*—selecting the proper meaning of an image; (2) *inflected words*—selecting the inflected word from an image; (3) *synonyms*—selecting the synonyms of two different words (in lower case) from two different words (in upper case); and (4) *word-meaning reading*—selecting the upper-case word which corresponds to the meaning based on various lower-case descriptions of the same concept. Test items of the instrument were structured into three sections. The opening module of the adaptive testing system was the same for all participants, and contained moderately challenging tasks. Based on their performance in the opening module, participants gained access to either an easy or a more difficult module. Acceptable achievement for the second level was set at 60%; those who could not reach it stayed on the first level. From the second level, acceptable achievement for the third level was set at 70%. On the third level, two different routes were possible depending on the participant’s performance level. Acceptable achievement for one route was set at 80%, and for the second one it was set at 90%.

#### 4.2.3. Musical Aptitude

The musical aptitude test consisted of 77 items in total (Surján and Janurik 2018). There were two different ways of grouping these items, which allowed for two different ways of creating the sub-test. One way was to create the musical-perception and the musical-reproduction sub-tests. The second way was to create the pitch-related-abilities (pitch perception and reproduction) and the rhythm-related-abilities (rhythm perception and reproduction) sub-tests.

*Musical Perception and Musical Reproduction*. In this arrangement, the instrument categorized musical skills—items—based on two aspects: (1) it measured the level of development of musical perception separately through listening discrimination tasks; and (2) that of musical reproduction with singing and clapping tasks.

The first section of the instrument measured the level of development of *musical perception* with discrimination tasks using tape-recorded materials (49 items). Except for the chord analysis and pitch discrimination II. tasks, participants had to decide whether two successive musical sound patterns they heard were the same or different. Task types included (1) melody discrimination: two successive short, sung melodies (7 items); (2) chord analysis: participants listened to a chord (consisting of two or three notes) played on the piano and had to tell the number of notes (8 items); (3) rhythm discrimination: two successive rhythmic patterns played on a snare drum (8 items); (4) pitch discrimination I.: two successive musical notes played on the piano (7 items); (5) tempo discrimination: short, simple piano excerpts (6 items); and (6) pitch discrimination II.: participants listened to two successive notes played on the piano and were required to decide if they were the same or different. They also had to determine the direction of pitch change (7 items) and (7) chord discrimination: two successive chords played on the piano (6 items). There was a 2.5 sec interval between the items of each item pair being compared.

The second section of the test consisted of *musical reproduction* tasks (28 items): (1) rhythm clapping: reproducing 2-beat-long rhythms by clapping (14 items); (2) interval-singing: reproducing voice-recorded intervals (7 items); and (3) melody-singing: reproducing voice-recorded, simple, 2-beat melodies (7 items).

*Pitch-related abilities and Rhythm-related abilities*. Grouping test items in a different way enabled us to examine *pitch-related and rhythm-related abilities* separately. Items measuring *pitch-related abilities* were as follows (49 items): (1) melody discrimination (7 items); (2) chord analysis (8 items); (3) pitch discrimination I. (7 items); (4) pitch discrimination II. (7 items); (5) chord discrimination (6 items); (6) interval-singing (7 items); and (7) melody singing (7 items). The *rhythm-related* sub-test consisted of the following tasks (28 items): (1) rhythm discrimination (8 items); (2) tempo discrimination (6 items); and (3) rhythm clapping (14 items).

The composite index of the *Musical-aptitude total test* included all test items.

#### 4.2.4. Music Reading

The music reading instrument was a paper–pencil test consisting of 6 tasks. It was based on the first-year curriculum of elementary schools, which relies on the Kodály method, and assessed students’ knowledge of solmization syllables and rhythmic-value symbols (name, symbol). Since musical note reading is introduced through teaching students the solmization syllables in the Hungarian education system, we hereinafter use the term “*solmization*” for musical note reading. Furthermore, we use the term “*rhythm reading*” for rhythm-related skills and knowledge.

The test battery consisted of 6 tasks measuring the development of rhythm reading and solmization (57 items). Rhythmic knowledge and solmization knowledge were assessed with 4 different tasks. One of these measured rhythm notations, while the other three measured pitch notation. However, it is difficult to distinguish between these two types of knowledge, as the music sheet contains rhythm-related as well as pitch-related information. Hence, the two types of notations were not distinguished in all 6 tasks. Instead, we included tasks that required the knowledge of both solmization syllables and rhythm. The instrument is, thus, capable of measuring the general level of development of music reading. We used seven different methods to examine the level of development of music reading.

*Rhythm reading*: (1) the standards of the first-year curriculum in relation to rhythm reading are typically met by students in Hungary, therefore, we decided to examine rhythm writing, which is based on one’s inner hearing and is considered as a difficult rhythm-reading task (task 1).

*Solmization:* (2) recognizing solmization syllables, five-line staff notation based on hand signs of solmization syllables (task 2a); (3) placing solmization syllables given in letter-name in five-line staff (task 3); and (4) naming the solmization syllables of a piece of sheet music in five-line staff (task 4a).

Further methods that relied on both *solmization* and *rhythm-reading*: (5) recognition based on inner hearing (task 2b); (6) copying notes of a five-line staff (task 5); and (7) transcribing musical sounds given in letter notation and rhythm on a five-line staff (task 6).

Tasks of the test are as follows:

(1) *Rhythm reading*: noting down a children’s song titled “*Hold-hold fényes lánc*”, which is well-known among Hungarian students and has quarter notes and eighth notes (8 items);
(2a) *Solmization*: recognizing and noting down solmization syllables given in hand signs (naming the solmization syllables—*sol, mi, do*—of a Hungarian children’s song titled “*Éliás, Tóbiás*” which are given in hand signs and rhythmic symbols, 8 items);
(2b) *Solmization* and *rhythm reading:* recognizing a song based on the solmization syllables and rhythmic symbols (1 item);
(3) *Solmization*: transcribing solmization syllables (mi, do, sol, la) given in letters on a five-line staff (4 items);
(4) *Solmization:* writing down the corresponding solmization syllables of the notes (d, e, h, g—sol, la, mi, do) given on a five-line staff without rhythm (12 items);
(5) *Solmization* and *rhythm reading*: copying notes transcribed on a five-line staff (16 items);
(6) *Solmization* and *rhythm reading*: musical staff notation of solmization syllables specified with rhythm (sol, mi, do) (8 items).

### 4.3. Procedure

Phonological awareness and word reading were measured on two separate occasions. The first assessment covered phonological awareness, followed by word reading on a separate day. We carried out online data collection using the eDia platform of the University of Szeged. Each data collection took 45 min. Students completed the tasks individually on PCs using headphones. Each student was provided with the same test in the same voice quality.

For the measurement of musical abilities, tablets were used. Students used headphones; instructions were given to them within the audio files. Students were allowed to listen to the audio instructions more than once, however, once they completed a task, they could not return to it. As for the reproduction tasks, students were assessed one by one in a quiet room. Each student listened to the same audio files, which contained pre-recorded singing and rhythm tapping, and the examiner recorded their reproductions. Students were allowed to listen to each audio file only once, and they were given only one chance per task for reproduction. Discrimination tasks took about 25 min; reproduction tasks took about 10 min per student.

The music reading test was administered during regular class hours; completion of the paper–pencil test took about 30 min. The instructions for the test were given by the examiner.

With the consent of parents, teachers completed a questionnaire to report the mothers’ level of education. Students were divided into three sub-samples according to parents’ level of education (elementary, secondary, higher). There were 5 students whose parents did not give their consent to include their level of education in the study. When interpreting the results, it is important to note that the vast majority of the students of the sample had mothers with secondary or higher level of education (elementary: *N* = 1, secondary: *N* = 60, higher: *N* = 53). According to the ratio of secondary and higher education sub-samples, there is no significant difference between the two groups (chi-squared = 0.43, *p* = .510).

### 4.4. Data Analysis

Data were analyzed with IBM SPSS Statistics, Version 19.0, as well as the RStudio program. We used descriptive statistics (means, standard deviations), Spearman bivariate correlations and partial correlation as well as step-by-step regression analysis. Results of the adaptive word reading test were calculated with the Rasch model. Means of musical abilities, music reading and phonological awareness tests were transformed into percentage values, range 0–100. Students’ ability level in word reading was established with the help of the Rasch model, then values of the logit units were converted into a scale, which was constructed to have a mean of 500 score points and a standard deviation of 100 score points.

## 5. Results

### 5.1. Preliminary Analysis

Table 1 shows the mean and standard deviation of the musical-aptitude total test as well as the values of the sub-tests, the level of development of music reading, word reading and phonological awareness and the number of items of tests and sub-tests as well as their reliability values.

### 5.2. Intercorrelations among Variables

We revealed significant correlations between musical aptitude (perception, reproduction, pitch-related abilities, rhythm-related abilities and total test), phonological awareness and word reading. The correlations of the mothers’ level of education with all the tests and sub-tests, except for music reading and word reading, were significant (Table 2).

Musical-aptitude total test showed a moderate correlation with word reading (*r* = .414, *p* < .001), a moderate/medium correlation with phonological awareness (*r* = .353, *p* < .001) and a significant, moderate correlation with music reading (*r* = .307, *p* < .001). We found strong correlations between musical sub-tests and the musical-aptitude total test (*r* = .742 and *r* = .936, *p* < .001). All musical sub-tests showed moderate/medium correlations with word reading: musical perception: *r* = .364 (*p* < .001); musical reproduction: *r* = .372 (*p* < .001); pitch-related abilities: *r* = .364 (*p* < .001); phonological awareness: *r* = .357 (*p* < .001); and rhythm-related abilities: *r* = .364 (*p* < .001). Musical perception had a moderate/medium correlation with phonological awareness (*r* = .364, *p* < .001) and a significant, moderate correlation with musical reproduction (*r* = .253, *p* < .001), pitch-related abilities (*r* = .292, *p* < .001) and rhythm-related abilities (*r* = .335, *p* < .001).

Mothers’ education showed a near significant correlation with word reading (*r* = .174, *p* = .064) and a significant, moderate correlation with phonological awareness (*r* = .325, *p* < .001). We established significant, low correlations between mothers’ education and musical sub-tests as well as the musical-aptitude total test (*r* = .222; *p* = .018, and *r* = .285; *p* = .002, respectively). We found a near significant correlation between mothers’ education and music reading (*r* = .169; *p* = .072).

Music reading moderately correlated with word reading (*r* = .604, *p* < .001) and a significant, moderate correlation with phonological awareness (*r* = .333, *p* < .001). Word reading also showed a moderate correlation with phonological awareness (*r* = .518, *p* < .001). Music reading showed a moderate/medium correlation with rhythm-related abilities (*r* = .378, *p* < .001), a moderate correlation with musical reproduction (*r* = .322, *p* < .001), and significant, low correlation with musical perception (*r* = .228, *p* = .012) and pitch-related abilities (*r* = .209, *p* = .023) (Table 2).

Phonological awareness and musical aptitude test as well as its sub-tests showed a significant correlation with word reading. Therefore, we calculated partial correlations between word reading and music reading controlling for phonological awareness and the musical-aptitude total. Again, the correlation between word reading and music reading was found to be moderate (*r* = .507, *p* < .001). After controlling for phonological awareness, the correlation was still significant between the musical-aptitude total and word reading (*r* = .289, *p* = .002). We found a significant, weak correlation between phonological awareness and musical perception (*r* = .216, *p* = .022), after controlling for word reading and mothers’ education.

### 5.3. Regression Analyses

We applied hierarchical, linear regression analyses to further explore the explanatory power of phonological awareness, musical aptitude (perception, reproduction, pitch-related abilities, rhythm-related abilities and total test) and music reading in text reading. Since mothers’ education did not show a significant correlation neither with text reading nor with music reading, we did not take this variable into consideration in our further analyses.

We tested the following models with step-by-step regression analysis: we used word reading as the dependent variable. Phonological awareness was entered in the first step. In the second step, we carried out three simultaneous analyses with different variables (analysis 2A, 2B and 2C). In analysis 2A, the musical-aptitude total was entered in the third step. In analysis 2B, musical perception and reproduction were entered in the third step. While, in analysis 2C, pitch-related and rhythm-related abilities were entered in the third step. In the third step, phonological awareness, musical-aptitude total and music reading were entered (Table 3).

According to the first model, the explanatory power of phonological awareness was 27% (*R*^2^ = .27, β = .52, *t*(1, 117) = 6.55, *p* < .001). In step 2A, the explained variance was 33% (*R*^2^ = .33) by entering the musical-aptitude total. Phonological awareness (β = .43, *t*(2, 116) = 5.23, *p* < .001) still showed the highest explanatory power with 22% (*r*β = .22), however, it decreased by 5%. The musical-aptitude total explained 11% of variance in word reading (β = .26, *t*(2, 116) = 3.25, *p* = .002). In step 2B, musical-perception and reproduction sub-tests were entered. Musical-reproduction skills showed significant explanatory power (*r*β = .08, β = .22, *t*(3, 115) = 2.31, *p* = .023), while musical perception did not (*r*β = .03, β = .08, *t*(3, 115) = 0.83, *p* = .407). Phonological awareness showed the highest explanatory power in the variance of word reading (*r*β = .22, β = .43, *t*(3, 115) = 5.30, *p* < .001). According to the analysis carried out in step 2C, pitch-related and rhythm-related abilities’ musical sub-tests did not explain significantly the individual differences in word reading (pitch-related abilities: *r*β = .06, β = .17, *t*(3, 115) = 1.91, *p* = .059; rhythm-related abilities: *r*β = .05, β = .15, *t*(1, 117) = 1.67, *p* = .099).

By entering music reading in step 3, the explained variance was increased with a further 17% (*r*β = .17, *R*^2^ = .50). Music reading showed the highest explanatory power, that is, 27% (*r*β = .27), in the variance of word reading (β = .45, *t*(3, 115) = 6.30, *p* < .001). Musical-aptitude total added a further, significant 7% (*r*β = .07) explanatory power (β = .17, *t*(3, 115) = 2.31, *p* = .023). Together, music reading and musical aptitude explained 34% (*r*β = .34) of the variance. In this model, the explanatory power of phonological awareness decreased to 16% (*r*β = .16, β = .31, *t*(3, 115) = 4.26, *p* < .001).

## 6. Discussion

We examined the relationship between word reading and music reading andmusical aptitude and phonological awareness, which is a key ability of reading. Our sample consisted of grade 1 elementary school students who were at the beginning of their reading and music reading studies. In fact, they started with their studies nine months before our assessment.

Results confirmed a link in the level of development of the two types of reading. We found a moderate correlation between the level of development in the music-reading total test and the word-reading test. We also found a moderate, significant correlation between the level of development in phonological awareness and reading, which is in line with previous results (Ziegler and Goswami 2005). Partial correlation between word reading and music reading was medium, even after controlling for phonological awareness and musical-aptitude total. Mothers’ education correlated with musical aptitude, musical sub-tests and phonological awareness. Furthermore, it showed a close-to-significant correlation with reading. The sample consisted of students’ whose mothers either had a secondary or higher level of education, except for one single student. Correlations showed that students whose mothers had a secondary level of education were at a disadvantage compared to students whose mothers had a higher level of education (e.g., Buckingham et al. 2013).

Step-by-step regression analysis showed that phonological awareness itself explained almost one-third of the individual differences in word reading. Including musical-aptitude total in step 2A added further significant explanatory power. Out of the musical sub-tests, musical reproduction was found to have statistically significant explanatory power. Adding music reading to the regression model, the independent variables explained 50% of the variance in word reading.

Based on previous research, variables of our regression model may not be considered as totally unrelated. Musical aptitude is associated with the development of phonological awareness and that of reading (Anvari et al. 2002; Butzlaff 2000; Degé and Schwarzer 2011). Our results have also confirmed a link between these skills and music reading. The predictive role of phonological awareness is widely accepted in the literature (Ziegler and Goswami 2005). However, our regression analysis results suggest that the skills under investigation have a significant predictive role in text reading.

The moderate/medium correlations we found between musical aptitude and its sub-tests and word reading as well as phonological awareness are consistent with the results obtained from previous studies. Musical activities may enhance the development of auditory perception and, along with it, speech perception. Some previous studies among students have confirmed the significance of rhythm perception in the development of phonological awareness and reading (e.g., David et al. 2007; Holliman et al. 2010; Moritz et al. 2013; Overy et al. 2003), while others have revealed the explanatory power of melodic or pitch discrimination (Barwick et al. 1989; Lukács and Hombolygó 2019). Forgeard et al. (2008b) found deficits in both the melodic- and rhythmic-discrimination abilities of dyslexic children. Our results suggest that the general level of development of musical perception and reproduction may play a role in the development of phonological skills as well as reading. We believe the differing results of previous studies can be explained by the differences in the musical-ability tests and intervention programs that have been used, the age of participants or the different music-education systems.

Our results may raise the possibility of a link between early music reading and early text reading. Both text reading and music reading are complex, multilevel activities that require to learn, use, and improve certain operations, skills, abilities and strategies, which are strongly associated (Forgeard et al. 2008a; Ziegler and Goswami 2005). According to Hansen and Bernstorf (2002), similar decoding skills are at play for music and text reading. Interpreting phonological awareness as an awareness and sensitivity to music makes it relevant in music reading too, along with phonemic awareness, if it is interpreted as an awareness towards the smallest units of musical sounds. Other reading-related skills such as sight identification (the instant recognition of words and musical notes etc., by glancing at them), orthographic awareness (the ability to understand the use of letters and musical symbols in written form), cueing systems awareness (the ability to gather meaning from information surrounding a word or musical phrase) and reading fluency (effortless, independent execution of text and music symbols; the ability to perform in a technically flawless manner) may also be compared to music reading (Hansen and Bernstorf 2002). Cantwell and Millard (1994) also suggest that processing staff notation has similarities with processing text. Our results indicate a link between the cognitive development that accompanies the acquisition of music reading and the cognitive development that accompanies text reading. For those students who are better in music reading, this may support early text reading. Our results relied on correlations and regression analyses. To the best of our knowledge, only one previous study has examined the development of beginners in text reading and music reading in this way, and it did not report such analyses. Betteney and Brooks (2015) conducted their investigation among 5–6-year-old children, and hypothesized that the early learning of music reading may have a transfer effect on the development of phonic-decoding skills. The significant correlations we found as well as the results of our regression analyses further reinforce the idea that a relationship between these two areas may in fact exist. Our results confirmed the widely accepted and important role of phonological awareness in the early development of text reading. However, our regression analysis suggests that the predictive role of music reading is even stronger than that of phonological awareness. A possible explanation for this may be that our music-reading variable represents a more complex, multilevel activity, while phonological awareness, one of the metalinguistic skills related to text reading, is a simpler skill compared to music reading (Ziegler and Goswami 2005). The other music-related variable we used in our model, that is, musical abilities, also has a significant relationship with both music reading and phonological awareness as well as with text reading. This may suggest that as a result of these relationships, the predictive role of phonological awareness in our model is represented in the explained variance of music reading. When interpreting our results in relation to text reading and music reading, the fact that these apply to the Hungarian language, which is a shallow orthographic language, is an important point to consider, as previous studies that focused on linguistic abilities and reading have typically involved English-speaking children. While the predictors of reading achievement are presumably universal in all languages and follow a similar developmental path, the orthographic consistency of a language influences the role phonological awareness plays in the development of reading, as the phonological structure of a spoken language affects certain metaphonological abilities (Ziegler et al. 2010). Moreover, the developmental characteristics of reading may also follow different paths. The more shallow, transparent and consistent the orthographic system is (and Hungarian is quite a transparent and consistent language), the shorter the first section of reading development is. These differences may have an effect on auditory processing mechanisms, coding and decoding mechanisms, their operation, the pace of development and, in line with this, musical auditory processing and the coding and decoding of musical notations.

According to Schellenberg and Weiss (2013), the direct facilitating role of music learning on the development of text reading cannot be established clearly. Rather, they assume that the two areas are connected through the development of phonological awareness as well as the ability to pronounce irregularly spelled words. Some previous studies found that children who engaged in musical-instrument learning had better reading skills, while other studies in the field examined the impacts of music programs, which often included learning to play a simple musical instrument. For example, Corrigal and Trainor (2011) measured reading comprehension among a sample of 6–9-year-old children with instrumental music training. According to their results, length of training was associated positively with reading comprehension when age, SES, music aptitude, IQ, word-decoding abilities and number of hours spent reading each week were controlled. It is important to emphasize that instrumental-music training requires the acquisition and continuous practice of music reading, and this is true for any music program that includes music reading or writing or other notation of musical sounds (e.g., Kodály-based programs). Therefore, when interpreting these results, the role of music reading (or any simple musical symbol reading), that is, learning to read music and its active role in the development of reading, should also be considered. Our results suggest that music reading, if taught simultaneously with text reading at an early stage of its acquisition, may act as a mediating factor that enhances the metacognitive components of reading acquisition as well as specific processes of auditory processing.

## 7. Conclusions

Our results confirm that the learning process of music- and text-reading support the development of specific cognitive functions that are used both in music and text reading. At the start of their learning process, the level of development of the two coding/decoding processes are related. Moreover, our findings suggest that the relations between music reading and text reading should be considered in planning future research and should also be considered in the interpretation of transfer-effect research. However, further research is needed to explore the background factors of the relationships identified as well as to examine the age characteristics in the link between text reading and music reading.

## Figures and Tables

**Table 1:** Descriptive statistics.

Tests, Sub-Tests	Number of Items	Cronbach’s α	Mean	SD
1. Phonological awareness	45	.86	61.16	17.70
2. Word reading	85	.96	526.35	78.26
3. Musical-aptitude total	77	.92	52.25	14.99
3.1.1. Musical perception	49	.79	59.65	14.01
3.1.2. Musical reproduction	28	.92	39.30	21.87
3.2.1. Pitch-related abilities	49	.91	49.27	17.84
3.2.2. Rhythm-related abilities	28	.81	57.41	16.17
4. Music reading	57	.91	89.96	11.90

**Table 2:** Intercorrelations of the variables.

Variables	1	2	3	4	5	6	7	8
1. Music reading	-							
2. Word reading	.604 **	-						
3. Phonological awareness	.333 **	.518 **	-					
4. Musical-aptitude total	.307 **	.414 **	.353 **	-				
5. Musical perception	.228 *	.364 **	.366 **	.901 **	-			
6. Reproduction	.322 **	.372 **	.253 **	.874 **	.576 **	-		
7. Pitch-related abilities	.209 *	.357 **	.292 **	.936 **	.875 **	.783 **	-	
8. Rhythm-related abilities	.378 **	.364 **	.335 **	.742 **	.607 **	.716 **	.459 **	-
9. Mother’s education	.169	.174	.325 **	.285 **	.222 *	.288 **	.236 *	.271 *

Note: N = 119; * Correlation is significant at the 0.05 level (2-tailed); ** Correlation is significant at the 0.01 level (2-tailed).

**Table 3:** Hierarchical regression analyses on word reading.

Independent Variables	R^2^	β	R^2^ Change	F	p
Step 1					
Phonological awareness	.27	.52	.27	42.89	<.001
Step 2A					
Phonological awareness		.43			
Musical-aptitude total	.33	.26	.06	28.48	<.001
Step 2B					
Phonological awareness		.43			
Musical perception		.08			
Musical reproduction	.33	.22	.06	19.23	<.001
Step 2C					
Phonological awareness		.42			
Pitch-related abilities		.17			
Rhythm-related abilities	.33	.15	.06	17.28	<.001
Step 3					
Phonological awareness		.31			
Musical-aptitude total		.17			
Music reading	.50	.45	.17	38.56	<.001

## Data Availability

Data are available upon request due to privacy restrictions.
